# Risk factors affect success rate of debridement, antibiotics and implant retention (DAIR) in periprosthetic joint infection

**DOI:** 10.1186/s42836-020-00056-2

**Published:** 2020-12-07

**Authors:** Yihong Xu, Liping Wang, Weidong Xu

**Affiliations:** Department of Osteoarthropathy Surgery, Changhai Hospital, Shanghai, 200433 China

**Keywords:** Risk factors, Success rate, DAIR, PJI

## Abstract

**Background:**

Periprosthetic joint infection (PJI) is the most devastating complication of joint replacement that seriously affects the quality of life and causes a heavy burden to the families and society. Due to shorter hospital stays, lower costs, improved joint function and less morbidity, a process of debridement, antibiotics and implant retention (DAIR) is recommended as the preferred treatment for acute periprosthetic joint infection. However, the factors that impact the success rate of DAIR remain controversial. This article evaluates the influential factors of DAIR and provides insights for orthopaedics surgeons to make optimal decisions to improve the success rate of DAIR.

**Conclusion:**

The poor general condition of patients, high preoperative C-reactive protein (CRP) level, repeated joint surgeries, and Methicillin-resistant *Staphylococcus aureus* (MRSA) infections may be associated with lower DAIR success rate. To the contrary, early surgery, radical debridement, exchange of removable components, washing with iodine and vacuum sealing drainage (VSD) may improve the success rate of DAIR. A sinus tract may not be absolutely contraindicated, but surgeons should treat it with caution. As there is no consensus on many issues, more high-quality research is required.

## Introduction

PJI is the most serious complication that contributes to more than a quarter of revision surgeries after joint arthroplasty [1, 2]. As people pay more attention to the PJI, the infection rate following arthroplasty is decreasing. However, the increase in the number of patients with prosthetic joints in recent decades has added to the absolute number of PJI cases [3, 4]. Recently, a review showed that, with more arthroplasty cases, the cost of revision surgery for infection has put greater pressure on healthcare budgets [5].

The treatment of PJI aims to eradicate infection, relieve pain and improve function. The therapeutic regimens include simple antibiotic treatment, DAIR, one-stage revision or two-stage revision, arthrodesis and amputation [2, 6]. Due to the presence of biofilm on the surface of the prosthesis, the success rate of antibiotic therapy alone is very low. This treatment is reserved for patients who refuse surgery, or who are unable to undergo surgery due to their clinical circumstances such as concomitant illness, fragility, or advanced age [7]. One- or two-stage revision arthroplasty often place a burden on patients and families because of its significant injury and high cost. Arthrodesis and amputation are usually the last choice in clinical practice because of serious impairment of joint function and quality of life. The accepted indications for arthrodesis or amputation include poor soft tissue conditions around the prosthesis and bone tissue defects, highly-resistant organisms, the patient’s poor general condition and failure of revision arthroplasty [8].

DAIR is currently considered the preferred treatment for acute PJI due to its low trauma, satisfactory cost-effectiveness and less impairment on joint function [2, 9, 10]. The infection remission rate of DAIR for hip and knee PJI ranges from 11.1 to 100% [9, 11]. However, improving the success rate of DAIR is still a challenge.

### Preoperative patient factors

#### Sinus tract

DAIR is only suitable for those PJI patients with good conditions of bone and soft tissue in whom no sinus tract exists between the joint prosthesis and the skin [12]. Qasim *et al* [13] showed that the emergence of a sinus tract was a high-risk factor for a failed DAIR procedure. Once the sinus tract forms, the success rate of DAIR is reduced greatly. Some scholars suggest that the emergence of a sinus tract is an absolute contraindication for DAIR.

With the development of a surgical technique for DAIR and the application of postoperative antibiotics, some studies showed that the existence of a sinus tract was no longer an absolute contraindication for DAIR. A retrospective study reported 14 PJI cases of a sinus tract treated with radical debridement, removal of dead tissue, shaving of pseudo-membrane, and intraoperative irrigation with a large volume of physiological saline, hydrogen peroxide and iodine. A satisfactory outcome was achieved in all patients as there was no recurrence of infection after 1–5 years follow-up [14].

#### Preoperative CRP level

Preoperative CRP level is also an influential factor contributing to a failed outcome of DAIR [15]. Vilchez *et al* [16] believed that DAIR achieved better results in PJI patients when preoperative CRP < 22 mg/L. However, Maritinez Pastor *et al* [17] believed that only when the preoperative CRP < 15 mg/L were outcomes better. Recently, a high-quality retrospective cohort study performed by Jacobs *et al* [18] reported that CRP level > 100 mg/L was associated with failure of DAIR. However, the cut-off value of CRP to help surgeons decide whether to perform the DAIR procedure cannot be determined at present. Therefore, the relationship between preoperative CRP level and the success rate of DAIR remains to be investigated further.

#### Repeated joint surgery

Previous joint surgery also affects the prognosis of DAIR. Abrman *et al* [19] reported that DAIR should be considered carefully in repeatedly operated joints. A multicenter cohort study performed by Peel* et al* [20] showed a higher risk of failure in cases of re-infection after revision surgery treated with DAIR.

#### Other factors

In addition to the above factors, age, immunity and nutritional conditions, the American Society of Anesthesiologists (ASA) score, and complications such as hypertension and diabetes also affect the success rate of DAIR [21].

There is no consensus on age. Although most scholars suggest increased age as a risk factor for failure, recently a high-quality meta-analysis [9] reported that the infection control rate was higher in older patients compared with younger patients. Moreover, a single-center retrospective study [22] reported that patients< 60 years old at the time of DAIR had a greater than two-fold increased risk of failure. Contradictory results relating age and DAIR success rate may be ascribed to the heterogeneity of studies, and further high-quality studies are required on this issue.

### Microbiology

Although a 100% success rate of DAIR for *Staphylococcus* infection has been reported [23], most studies reported a higher failure rate of DAIR when performed to treat *Staphylococcus* infection [11, 22, 24–26]. A multicenter observational study performed by Weston *et al* [22] reported a three-fold increased risk of failure after DAIR in cases of *Staphylococcus* infection. Wouthuyzen *et al* [25] showed that although the outcome of DAIR in the management of early acute PJI was satisfactory, its use in late acute PJI caused by *Staphylococcus* should be reconsidered. A recent study found that the success rate in patients treated with DAIR for *Staphylococcus* infection was 76.2% [26].

The success rate of DAIR for treating periprosthetic infection caused by *Streptococcus* is relatively high [27, 28]. Lam *et al* [27] reported that the initial success rate of DAIR in the treatment of 53 PJI cases caused by *Streptococcus* infection was 84%. However, in 2017, a large multicenter retrospective study showed that the success rate of DAIR for *Streptococcus* infection was only 57.9% [29].

Periprosthetic infections caused by gram-negative bacteria have been infrequently reported. A large multicenter retrospective observational study [30] reported a 79% success rate for gram-negative prosthetic joint infection treated with DAIR.

McArthur et al [31] reported 16 of 26 infections caused by organisms of low virulence such as Coagulase-negative *Staphylococcus*, *Acinetobacter*, *Propionibacterium* species, and *Corynebacterium* species which were more common in seronegative infections. This study suggested that low virulence infections may contribute to a relatively high success rate. However, the sample size was small and further data are needed.

### Timing of the surgery

Fehring *et al* [32] showed a limited ability of DAIR to control infection in the early postoperative period following arthroplasty. Grammatopoulos *et al* [28] suggested DAIR be performed in all PJI cases, regardless of interval from the index symptoms. A similar PJI eradication rate was seen if DAIR was undertaken after six (78%) or 13 weeks (83%). However, most scholars regard the timing of DAIR as a key factor in its success rate in PJI patients. In general, DAIR is recommended for acute infection or acute hematogenous infection.

Sendi *et al* [33] reported a successful outcome rate in 91% of 34 PJI cases after total hip arthroplasty (THA), the duration of symptoms did not exceed 21 days. Ottesen *et al* [34] reported that there was no obvious difference in success rate between DAIR performed within 28 (85%) or 42 days (88%). However, the success rate was significantly lower when performing DAIR after 90 days (60%). A review of cohort studies that included 1296 patients [35] reported a statistically significantly greater success rate when DAIR took place at a median of less than 7 days from the onset of symptoms of infection compared with debridement performed at a median of 1 week after the onset of symptoms (72.0% versus 51.8%).

The presence of biofilm on the surface of an implant is the most important reason for the transformation of an early infection of an artificial joint into a chronic infection [36]. Three weeks after infection, pathogenic microorganisms form a membranous structure on the surface of the prosthesis and enter the maturation stage. At this point, even open debridement can’t remove the biofilm and bacteria sessiled in the biofilm completely. Meanwhile, due to the continuous shedding of biofilm debris, bacteria in the biofilm become plankton, resulting in continuous and repeated infection. Above all, DAIR can be used to treat patients with acute PJI within 4 weeks of infection before the bacteria have been sessiled on the surface of the prosthesis and the biofilm matures. Therefore, we believe that DAIR should be performed as soon as possible after the identification of symptoms of acute infection to avoid the transformation of an acute infection into a chronic infection.

### Arthroscopic or open debridement

Liu *et al* [37] reported an 88% success rate in 15 patients treated by arthroscopic debridement and continuous antibiotic irrigation. Dixon *et al* [38] showed that arthroscopic debridement was more effective in patients with periprosthetic infection of the knee. Though findings of a meta-analysis [23] suggested a higher infection control rate for arthroscopic debridement compared with open debridement, the results were not significantly different. The pooled infection control rate for arthroscopic DAIR was based on only a few studies. More studies showed a lower success rate of arthroscopic DAIR when compared to open debridement [39, 40]. We suggest the DAIR procedure cannot be carried out arthroscopically because it does not allow adequate debridement or exchange of the polyethylene liner.

### Radical debridement

Thorough debridement is essential to the success of DAIR. All infected tissues, synovial membrane, necrotic tissues, scar tissue and foreign materials must be completely removed. The success rate of DAIR in PJI patients is very low if debridement is not performed [41].

### Exchange of removable components

The prosthesis is retained when DAIR is performed to treat PJI, but removable components must be exchanged whenever possible [13, 28, 35, 42]. T Sang *et al* [35] reported a mean success rate of 73.9% in PJI patients who underwent exchange of modular components (femoral head or acetabular liner) at the time of debridement. This was higher than in patients for whom no components were exchanged (60.7%). Recently, a single-center retrospective cohort study performed by Hirsiger *et al* [42] showed that the exchange of mobile components of the PJI during DAIR almost doubled the long-term remission rate compared to the control group. Almost all removable components are made of polyethylene to which bacteria can easily adhere and some bacteria remain in the dead corner and the back of the liner after debridement. Only by exchanging components can we debride the infection thoroughly. Once the components are retained during debridement, the infection may recur easily and this ultimately can lead to the failure of treatment.

### Washing with povidone-iodine

High volume povidone-iodine and low-pressure irrigation should be used intraoperatively with a lavage volume at least 9 L. Li *et al* [43] reported that the success rate of washing with iodine in DAIR was 89.3%. They suggested that for patients with PJI within 4 weeks, DAIR could be performed. When the prosthesis was soaked with iodine four times intraoperatively, the curative effect was better than conventional debridement. High-volume irrigation of povidone-iodine intraoperatively similarly was recommended by Choo KJ *et al* [44]

### Vacuum sealing drainage (VSD)

VSD helps to continuously drain necrotic tissue and exudate, eliminate edema, stimulate growth of the granulation tissue, improve local blood supply and enhance local immunity. Since its development, the VSD technique has been applied to the treatment of PJI with increasing frequency.

The negative pressure value of VSD also had a significant influence on the effect of drainage. When high negative pressure drainage was performed, the total drainage and infection control rate were significantly better compared to low negative pressure drainage [45]. In 2014, Anagnostakos *et al* [46] reported that the success rate of DAIR combined with VSD was as high as 92.8%.

We suggest VSD be used for selected patients because of limitations such as requirement for frequent exchange of materials which increases hospitalization expenses and hospital stays.

### Single or multiple debridement

A voluminous literature discussed the relationship between single or multiple debridement and the success rate of DAIR. Some scholars reported that multiple debridement was related to the increased failure rate of DAIR [42, 47–50]. Estes* et al* [47] reported that 18 of 20 PJI patients utilizing a two-stage debridement technique with the implantation of antibiotic cement beads in stage one achieved a satisfactory outcome. Geurts *et al* [48] also reported good results for deep infections following total hip or knee arthroplasty with multiple debridement. Romano *et al* [49] reported that the success rate of multiple debridement was higher than that of single debridement. Chung AS *et al* [50] reported that 2-stage debridement led to a greater likelihood of infection control compared to single stage debridement. In contrast, a case control study by Moojen *et al* [51] reported a retrospective comparative study between single debridement and multiple debridement, finding no statistically significant difference in the proportion of successful outcomes between the two groups (88% *vs.* 71%). Moreover, Jacobs *et al* [18] found that multiple debridement procedures contributed to failed DAIR due to the risk of joint and wound contamination during the surgery. As there is no consensus, we suggest that orthopedists should be meticulous when performing multiple debridement, considering the length of the hospital stay, cost and morbidity associated with failed DAIR.

### Postoperative antibiotics

When PJI patients were treated by DAIR without postoperative antibiotics, the failure rate after 3 years follow-up was as high as 86% [36].

For PJI patients with positive bacterial culture and drug sensitivity test results, antibiotics sensitive to pathogenic bacteria should be used after DAIR. The selected antibiotics also should be biofilm- penetrative to kill the pathogenic bacteria remaining in the biofilm after DAIR.

For PJI patients with negative bacteria culture or no drug sensitivity results, empirical antibiotic therapy usually is required. Selected antibiotics must have an adequately broad antibacterial spectrum to cover common pathogenic microorganisms. Sousa *et al* [52] suggested that vancomycin could be selected for patients with culture-negative PJI. However, Aboltins *et al* [53] reported that postoperative use of rifampicin combined with ciprofloxacin for culture-negative PJI led to a good outcome. The international consensus on PJI published by Parvizi *et al* [54] in 2013 suggested that vancomycin should be used for gram-positive bacteria and gentamicin or the third and fourth generation cephalosporin should be used for gram-negative bacteria in areas where MRSA is prevalent. However, for areas with low MRSA prevalence, the empirical use of vancomycin was not recommended until the result of drug sensitivity is available.

As to the duration of antibiotic treatment after DAIR, the Infectious Diseases Society of America (IDSA) advises initial intravenous therapy for 2–6 weeks followed by oral antibiotics for 3 months after hip surgery or 6 months after knee surgery. Several studies showed no differences in infection control rate when comparing shorter duration with longer duration postoperative antibiotics. Other studies indicate that prolonged parenteral antibiotic therapy increases the economic burden and the risk of drug resistance [55, 56].

## Conclusion

DAIR is the preferred treatment for acute PJI with lower trauma and cost compared to revision surgery. A poor general condition of the patient, high preoperative CRP level, repeated joint surgery, MRSA infections may be associated with reduced DAIR success rate. Early surgery, radical debridement, the exchange of removable components, washing with iodine and VSD may improve the success rate of DAIR. A sinus tract may not absolutely contraindicate DAIR, but surgeons should treat it with caution. Orthopedic surgeons should take precautions to achieve a better outcome in PJI patients. As studies about DAIR are mostly retrospective and involve small samples, there is still no consensus on many aspects of DAIR effectiveness. More high-quality researches are required to improve the success rate of DAIR for PJI.

## Data Availability

Data sharing not applicable to this article as no datasets were generated or analyzed during the current study.

## Abbreviations

PJI: Periprosthetic joint infection
DAIR: Debridement, antibiotics and implant retention
CRP: C-reactive protein
ASA: American Society of Anesthesiologists
MRSA: Methicillin-resistant *Staphylococcus aureus*
THA: Total hip arthroplasty
VSD: Vacuum sealing drainage
IDSA: Infectious Diseases Society of America
